# Supplementary material for: Glyphosate is a transformation product of a widely used aminopolyphosphonate complexing agent
Source: Nat Commun. 2025 Mar 11;16:2438. doi: 10.1038/s41467-025-57473-7 (PMC11897348; doi:10.1038/s41467-025-57473-7)
Supplement: Supplementary file 1 — Supplementary Information [file 41467_2025_57473_MOESM1_ESM.pdf]

Supplementary information to:

Glyphosate is a transformation product of a widely used aminopolyphosphonate complexing agent

Anna M. Röhnelt[1], Philipp R. Martin[1,5], Mathis Athmer[2], Sarah Bieger[3], Daniel Buchner[1], Uwe Karst[2], Carolin Huhn[3], Torsten C. Schmidt[4] & Stefan B. Haderlein[1*]*

[1]Geo- and Environmental Research Center, Department of Geosciences, University of Tübingen, Tübingen, Germany

[2]Institute of Inorganic and Analytical Chemistry, University of Münster, Münster, Germany

[3]Institute of Physical and Theoretical Chemistry, Department of Chemistry, University of Tübingen, Tübingen, Germany

[4]Instrumental Analytical Chemistry and Centre for Water and Environmental Research (ZWU), University of Duisburg-Essen, Essen, Germany

[5]Current address: Division of Environmental Geosciences, Centre for Microbiology and Environmental Systems Science, University of Vienna, Vienna, Austria

[*]Corresponding authors: philipp.martin@univie.ac.at, stefan.haderlein@uni-tuebingen.de

Pages: 16
Figures: 12
Tables: 7

RESULTS AND DISCUSSION

pH control in conducted experiments

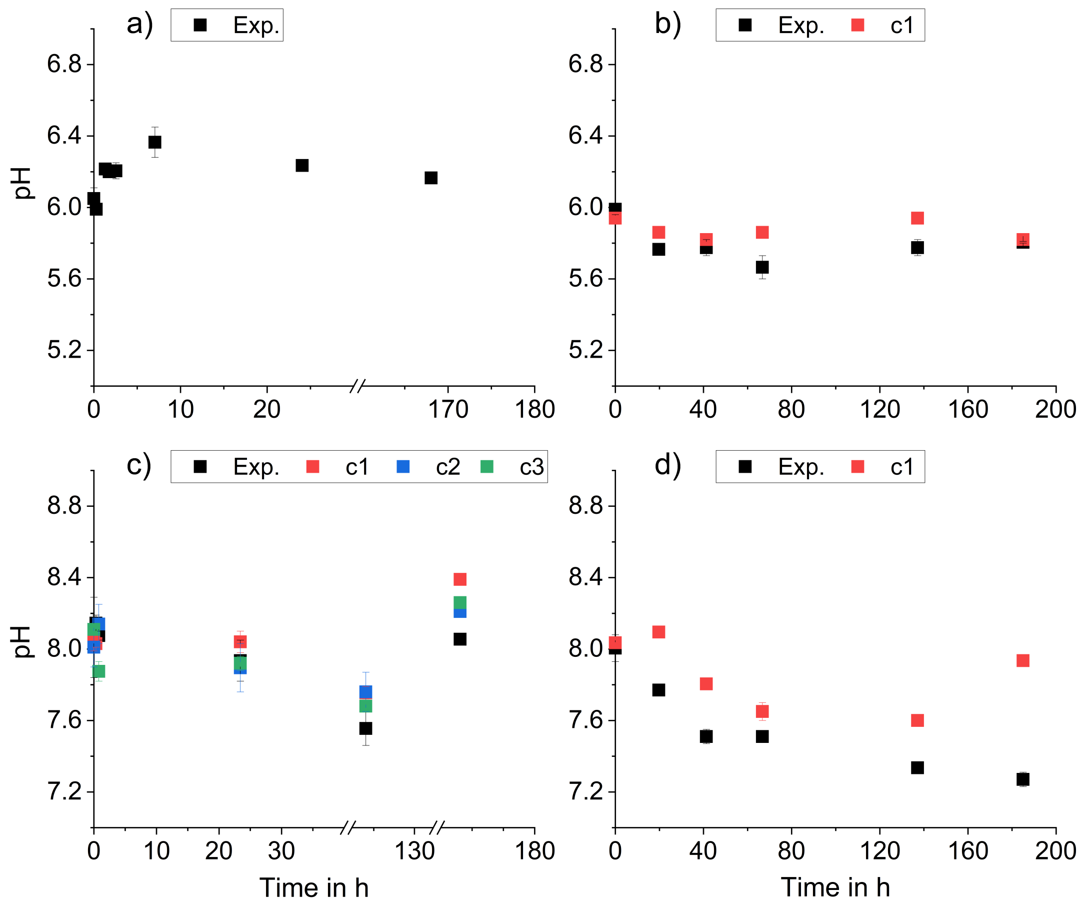

Fig. S1: pH values over time in four different DTPMP transformation experiments (Exp.) including several controls (c1-c3, respectively). a): Exp.: 1.0 g/L MnO_2 oxic in MES (black); b): Exp.: 1 mM $MnCl_2$ oxic in MES (black), c1: no $MnCl_2$ (red); c): Exp.: 1.0 g/L MnO_2 oxic in wastewater (black), c1: pure wastewater (red), c2: no MnO_2 (blue), c3: no DTPMP (green); d): Exp.: 1 mM $MnCl_2$ oxic in wastewater (black), c1: no DTPMP (red).

DTPMP stability in the absence of manganese

In the absence of MnO_2 and Mn^{2+} no DTPMP transformation and no AMPA or glyphosate formation were detected.

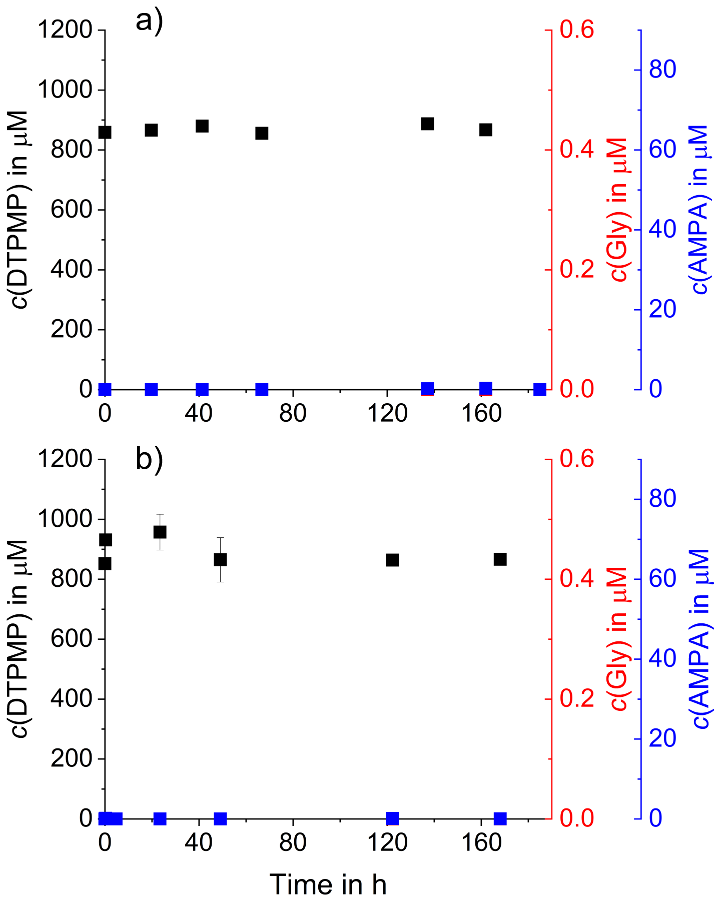

Fig. S2: DTPMP (black), glyphosate (red) and AMPA (blue) concentrations quantified by means of IC-IPAD (DTPMP) and LC-QQQ (glyphosate, AMPA) in two control experiments without manganese in a) 20 mM MES buffer (pH 6) and b) wastewater (pH 8). DTPMP was quantified using IC-IPAD, while glyphosate and AMPA were quantified using LC-QQQ.

DTPMP transformation in the presence of MnO_2 and 1 mM Mn^{2+} at pH 6

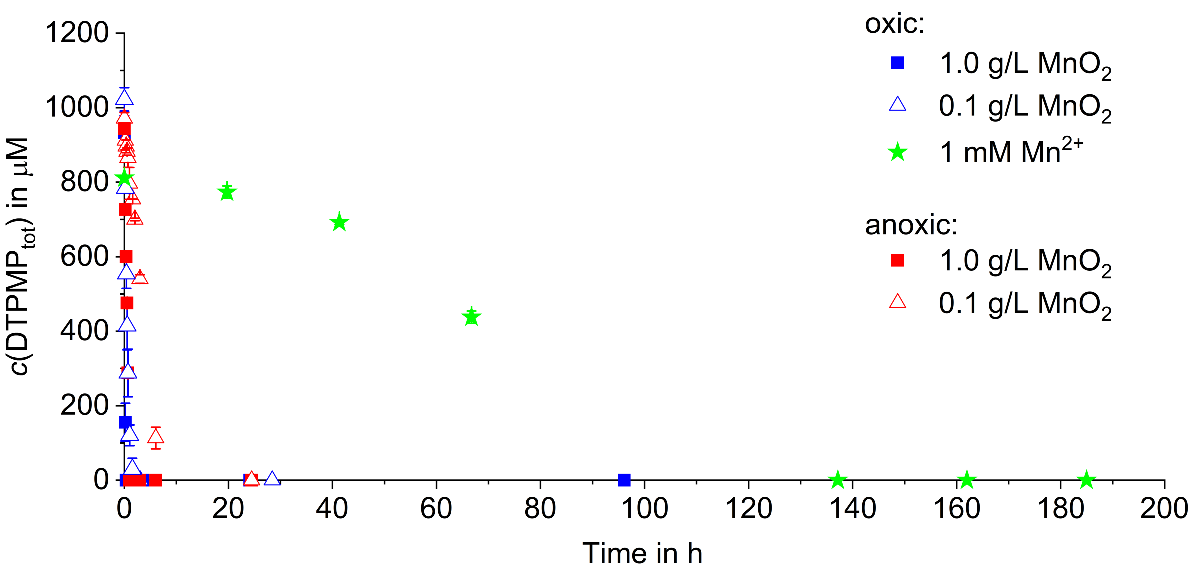

Fig. S3: Total DTPMP concentrations quantified using IC-IPAD as a function of time for all four experiments with MnO_2 (oxic (blue) and anoxic (red)) and one experiment containing 1 mM $MnCl_2$ (oxic, green) in aqueous MES buffer (pH 6). (Figure 2 in the main text amended by the experiment containing 1 mM Mn^{2+} (oxic conditions) at pH 6.) Error bars represent absolute errors between duplicates.

Additional concentration profiles of transformation experiments

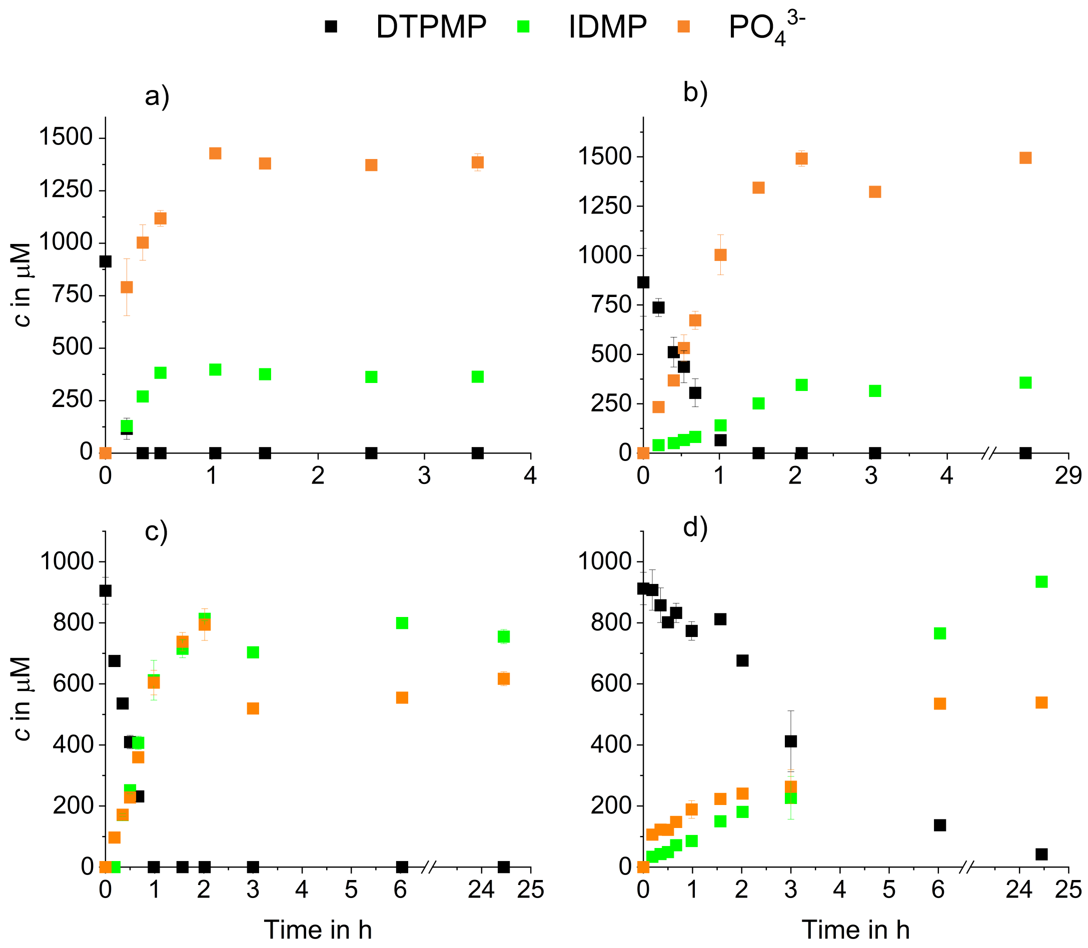

Fig. S4: Aqueous DTPMP (black), IDMP (green) and PO_4^{3-} (orange) concentrations quantified using IC-ICP-MS ($^{31}P^{16}O^{+}$) during DTPMP oxidation by MnO_2 in four different experiments. a) 1.0 g/L MnO_2 oxic, b) 0.1 g/L MnO_2 oxic, c) 1.0 g/L MnO_2 anoxic, d) 0.1 g/L MnO_2 anoxic. Error bars represent absolute errors between experimental duplicates.

Long-time replica of 1.0 g/L MnO_2 in MES buffer

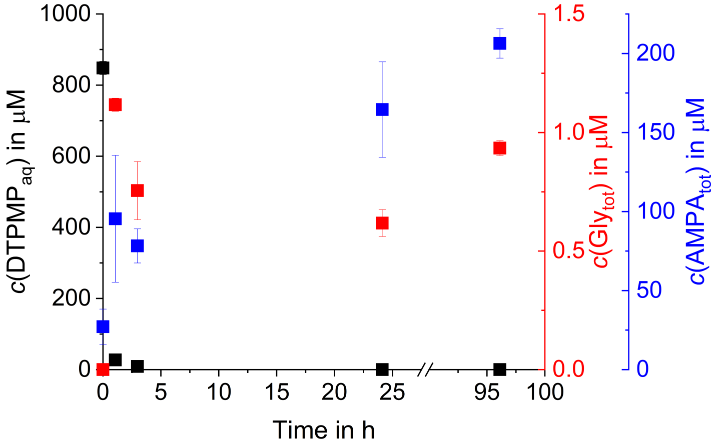

Fig. S5: Aqueous concentrations of DTPMP (back) quantified by means of IC-ICP-MS and total concentrations of glyphosate (red) and AMPA (blue) quantified by means of LC-QQQ in the longtime replica of the experiment using 1.0 g/L MnO_2 in ultrapure buffered water (pH 6) under oxic conditions over 96 hours. Error bars represent absolute errors between experimental duplicates.

AMPA and glyphosate in the DTPMP-free wastewater controls

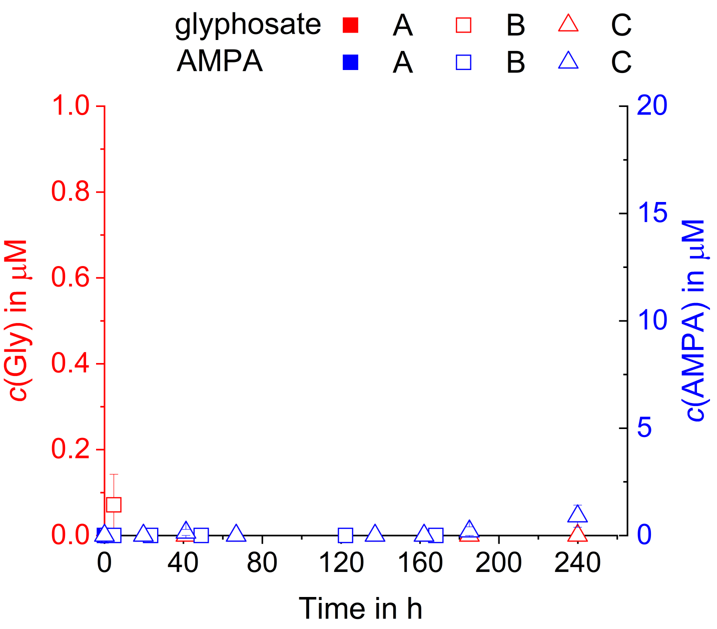

Fig. S6: Glyphosate (red) and AMPA (blue) concentrations quantified by LC-QQQ in control experiments without the addition of DTPMP. A: pure wastewater, B: wastewater with 1.0 g/L MnO_2, C: wastewater with 1 mM $MnCl_2$. Note: Due to data point overlay, not all individual data points are visually distinguishable.

DTPMP transformation by MnO_2 in wastewater vs. MES buffer

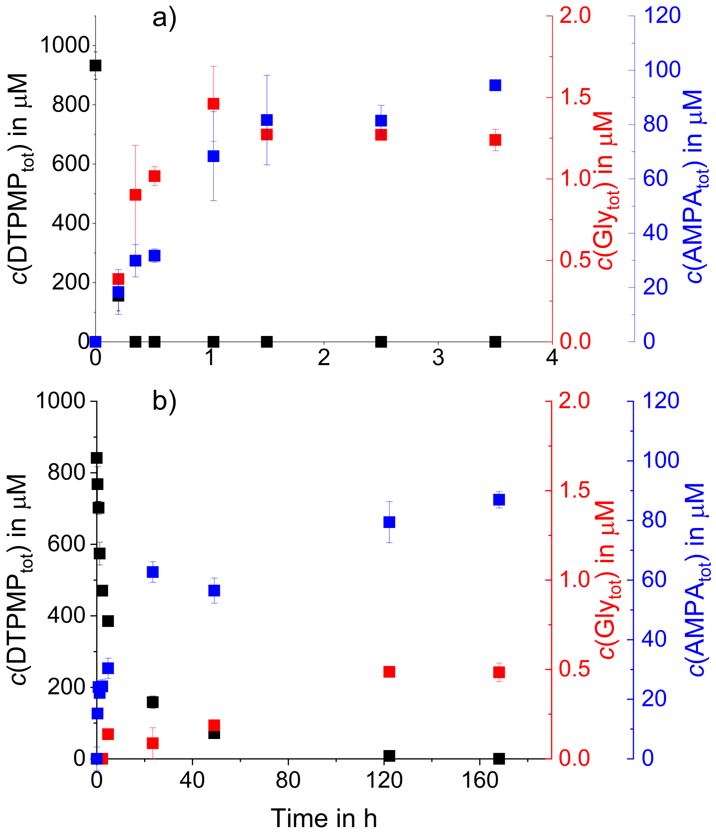

Fig. S7: Total DTPMP (black), glyphosate (red) and AMPA (blue) concentrations quantified by means of IC-IPAD (DTPMP) and LC-QQQ (glyphosate, AMPA) in two experiments containing 1 mM DTPMP and 1.0 g/L MnO_2 in a) 20 mM MES buffer (pH 6) and b) wastewater (pH 8).

METHODS

Quantification of DTPMP

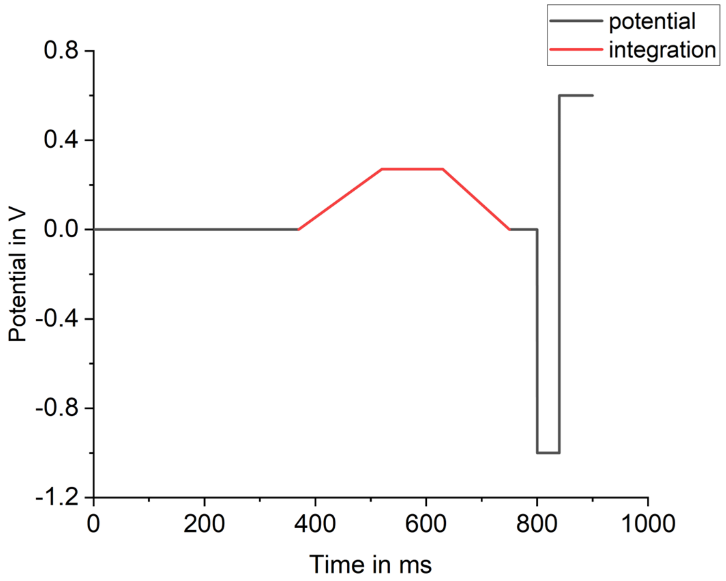

Fig. S8: Detector method or "waveform" for the amperometric detection used in the IC-IPAD method.

Table S1: Concentration gradient profile of 15 mM NaOH (eluent A) and 50 mM NaOH and 400 mM NaAc (eluent B) for the quantification of DTPMP by IC-IPAD.

Time in min	Share eluent A in %	Share eluent B in %
0.0	100	0
7.1	90	10
15.1	70	30
19.0	0	100
20.0	0	100
20.1	100	0
31.0	100	0

Quantification of AMPA and glyphosate

Table S2: Concentration gradient profile of 2.5 mM aqueous ammonium acetate (eluent A) and acetonitrile (eluent B) for the quantification of AMPA and glyphosate by LC-QQQ.

Time in min	Share eluent A in %	Share eluent B in %
0.0	95	5
2.0	95	5
8.0	30	70
8.5	0	100
13.0	0	100
13.1	95	5
16.0	95	5

Table S3: MS/MS parameters for the quantification of AMPA and glyphosate by LC-QQQ. All compounds were derivatized using FMOC-Cl.

Compound	Precursor ion (m/z)	Product ion (m/z)	Fragmentor in V	Collision energy in eV	Retention time in min
Glyphosate	392	179	100	24	6.15
	392	88	100	16	
$^{13}C^{15}_2N$– Glyphosate	395	179	100	24	6.15
	395	91	100	16	
AMPA	334	179	100	11	7.03
	334	112	100	10	
$^{13}C^{15}N$– AMPA	336	179	100	11	7.03
	336	114	100	10	
Glufosinate	404	179	100	24	6.42
	404	136	100	20	

The following table holds LOD/LOQ values for AMPA and glyphosate LC-QQQ measurements derived as stated in the Methods section of the main text.

Table S4: LOD and LOQ values for glyphosate and AMPA in µg/L for each measurement sequence for the LC-QQQ measurements. The approach used to derive those LOD/LOQ values is described in the Methods section. The controls are displayed below the respective experiment.

Experiment			Glyphosate		AMPA	
**Manganese**	**O_2**	**Matrix**	**LOD**	**LOQ**	**LOD**	**LOQ**
1.0 g/L MnO_2	oxic	MES	10.48	17.44	6.41	10.03
0.1 g/L MnO_2	oxic	MES	5.09	12.13	6.31	10.73
1.0 g/L MnO_2	anoxic	MES	9.80	33.47	9.49	20.94
0.1 g/L MnO_2	anoxic	MES	3.57	6.98	9.49	20.94
1 mM Mn^{2+}	oxic	MES	4.63	8.64	4.42	8.94
– control 1			4.63	8.64	4.42	8.94
1.0 g/L MnO_2	oxic	MES	4.89	10.14	4.41	8.94
– control 1			3.57	6.98	1.17	4.83
– control 2			5.50	11.00	5.36	12.04
– control 3			3.57	6.98	1.17	4.83
1 mM Mn^{2+}	oxic	MES	4.63	8.64	5.36	120.04
– Control 1			4.63	8.64	4.42	8.94

Aqueous and sorbed fractions of AMPA and glyphosate

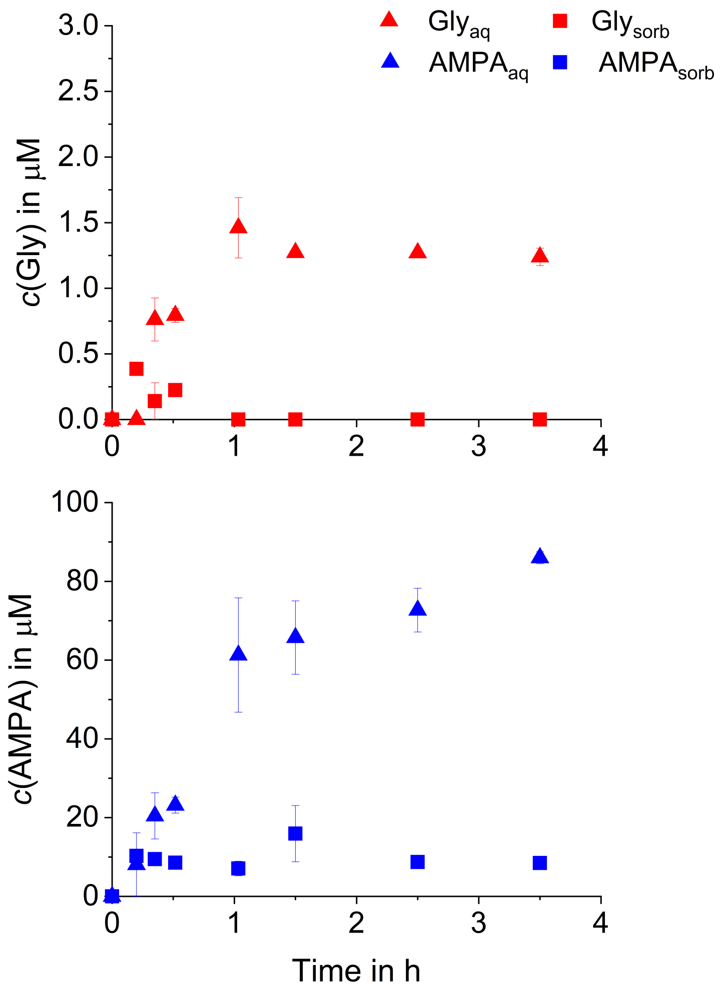

Fig. S9: Aqueous and sorbed glyphosate (red) and AMPA (blue) concentrations quantified by means of LC-QQQ in the experiment with 1.0 g/L MnO_2 under oxic conditions (pH 6). Error bars represent absolute errors between experimental duplicates.

Quantification of phosphorus-containing TPs using IC-ICP-MS

Table S5: Concentration gradient profile of 300 µg/L aqueous DTPA (pH 9.2) (eluent A) and 150 mM aqueous ammonium nitrate with 300 µg/L DTPA (pH 9.2) (eluent B) for the quantification of P-containing compounds by IC-ICP-MS.

Time in s	Share eluent A in %	Share eluent B in %
0	91.5	8.5
25	86.5	13.5
120	60.0	40.0
155	30.0	70.0
185	12.0	88.0
245	12.0	88.0

Nuclear Magnetic Resonance Spectroscopy (NMR)

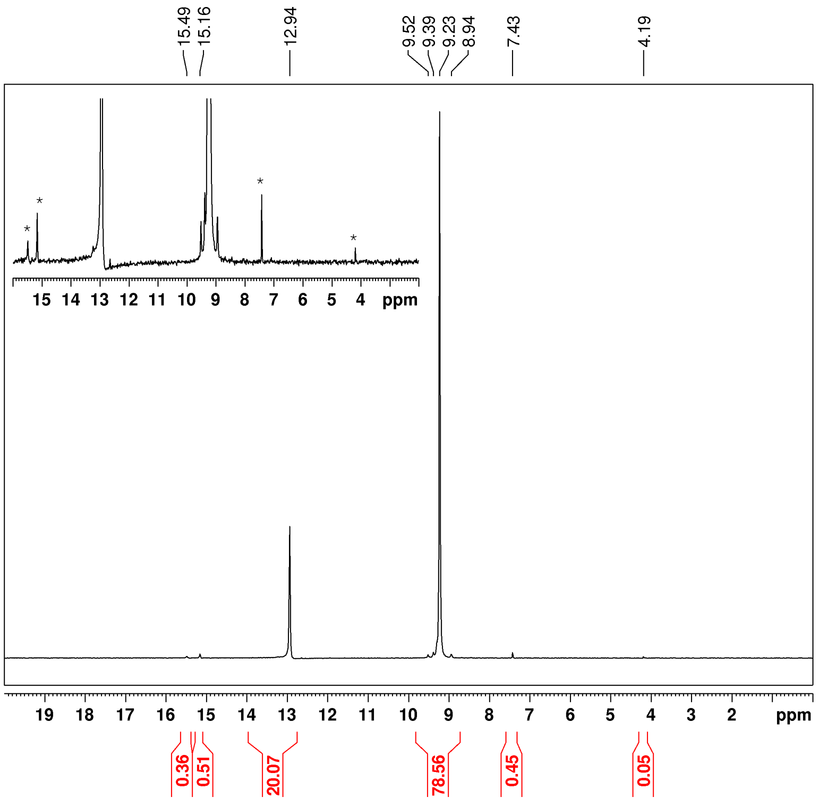

Fig. S10: ^{31}P–^{1}H-NMR-spectrum of DTPMP in D_2O measured as stated in the Methods section. δ (ppm): 9.23, 12.94. Impurities are marked with an asterisk. The sum of integrals is normalized to 100.

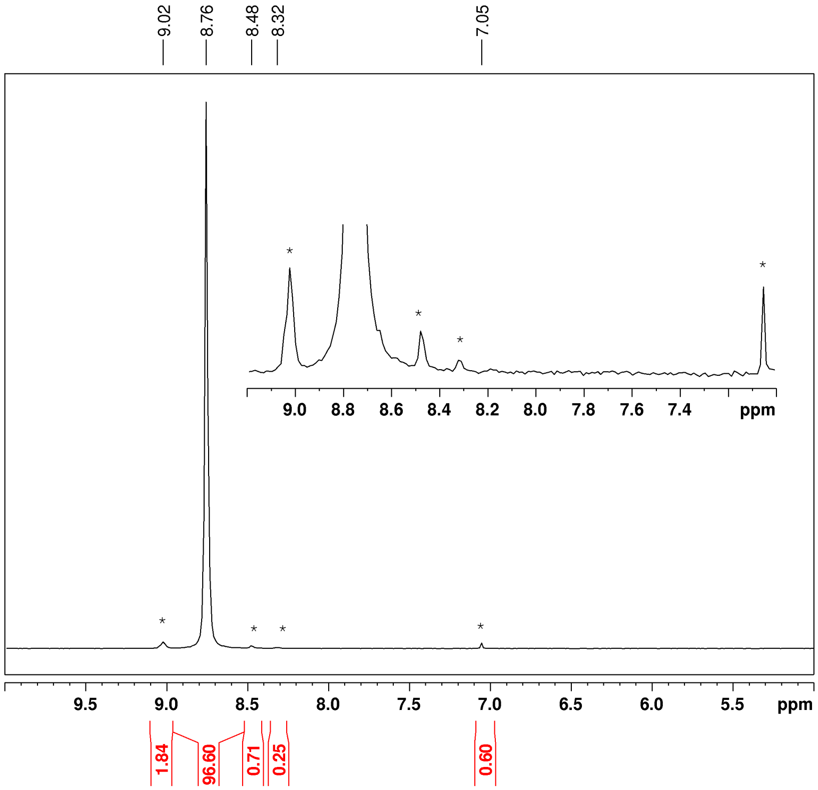

Fig. S11: ^{31}P–^{1}H-NMR-spectrum of EDTMP in D_2O measured as stated in the Methods section. δ (ppm): 8.76. Impurities are marked with an asteriks. The sum of integrals is normalized to 100.

Mineral Characterization

Point of zero charge (pH_{PZC})

The pH_{PZC} of the MnO_2 used in this study was analyzed via ζ-potential measurements using a Zetasizer Nano ZSP (Malvern Pananalytical, Malvern, United Kingdom) in folded capillary zeta cells at 20 °C. Measurements were conducted in triplicates with 10 to 15 runs, each. For analysis, 50 mg/L MnO_2 suspensions were prepared in 10 mM NaCl + 10 mM MES buffer and the pH was adjusted by 0.1 M or 1 M NaOH and HCl. The point of zero charge (pH_{PZC}) was determined by plotting the zeta potential as a function of the adjusted pH and subsequent regression of the linear part of the data sets. pH_{PZC} was determined to be at 5.6 ± 0.1.

Brunauer-Emmett-Teller method (BET)

The specific surface area (SSA) of the MnO_2 was determined by nitrogen sorption-desorption isotherms using a Gemini VII 2390 (Micrometrics, Norcross, GA, USA). Samples were degassed before analysis overnight under vacuum at 120 °C. SSA was determined at 64.5 ± 0.2 m^2/g.

Powder X-ray diffraction (XRD)

XRD measurements were performed in Göttingen by Volker Karius using an Orion Comet P2 Powder diffractometer (XRD Eigenmann GmbH, Schneittach-Hormersdorf, Germany) equipped with a Cu–source (Cu-Kα radiation, $\lambda_1 = 1.54060$ Å, $\lambda_2 = 1.54443$ Å) with a $K\alpha_2/K\alpha_1$ relation of 0.5 and a beam voltage and current of 40 kV and 40 mA, respectively. The mineral predominantly exhibits an amorphous structure interspersed with localized crystalline domains consisting of the two polymorphs pyrolusite (β-MnO_2) and akhtenskite (ε-MnO_2).

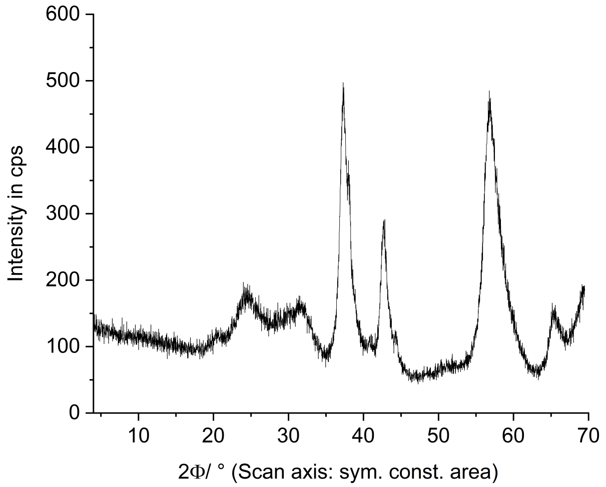

Fig. S12: X-ray diffractogram of the manganese dioxide used in this study. Measurement details are given in the text above.

Further mineral analysis

The extractable Mn^{III}-content (quantified by pyrophosphate extraction and UV/vis spectroscopy) was 1% of total manganese. With regard to the surface area, the extractable Mn^{III}-content was 1.76 nmol/m^2. Further, total reflection X-ray fluorescence (TXRF) revealed just minor amounts of mono- and bivalent cations (K: < 0.001, Ca and Ba: ≤ 003 molar ratio vs Mn), which validates the low Mn^{III}-content.

Wastewater Characterization

Inorganic cation and anion analysis was performed using ion chromatography coupled to conductivity detection (CD). The ion chromatograph (930 Compact IC Flex) was equipped with a 919 IC Autosampler plus and two 800 Dosinos (all Metrohm, Filderstadt, Germany) for automated sample dilution and eluent production. On the anion side, a suppressor (MSM Rotor A, Metrom) was installed prior to the detector. The column Metrosep A Supp 5 (4.0 x 250.0 mm) was employed for anion analysis, while the Metrosep C6 (4.0 x 250 mm) was employed for cation analysis (both Metrohm). The eluent on the anion side consisted of 3.2 mM sodium carbonate and 1.0 mM sodium bicarbonate in ultra-pure water and was delivered at a flow rate of 0.7 mL/min. The eluent on the cation side consisted of 4.0 mM nitric acid and 0.7 mM dipicolinic acid delivered at a flow rate of 0.9 mL/min. The injection volume was 20 µL.

Dissolved organic carbon (DOC) was analyzed using a vario TOC cube (Elementar, Langenselbold, Germany). Prior to analysis, the samples were filtered (0.45 µm), acidified to a pH of less than 2 using 32% HCl and purged with synthetic air to remove inorganic carbon. The analysis was conducted in duplicates.

Aqueous manganese and iron in the filtered wastewater sample was quantified by a 4200 MP–AES, equipped with a SPS 3 autosampler, a cyclonic spray chamber and an easy–fit torch (Agilent Technologies, Santa Clara, United States). The selected wavelengths were 403.076 nm (Mn) and 373.486 nm (Fe). Samples were diluted prior to analysis 1:2, with a final concentration of 1% (v/v) HNO_3. External standards in the range between 0.01 mg/L and 5.0 mg/L were used for quantification ($R^2 > 0.999$). LOD for Mn was determined to be 0.038 mg/L, while the LOD for Fe was determined to be 0.019 mg/L.

Table S6: Anions and cations quantified in the wastewater sample using ion chromatography with conductivity detection, except for Mn and Fe, which were quantified using microwave-plasma atomic emission spectroscopy (MP-AES), and therefore are not assigned cationic charges.

**Anion**	***c* in mg/L**	**Cation**	***c* in mg/L**
F^-	0.23	Na^+	27.9
Cl^-	39.1	NH_4^+	13.0
NO_2^-	0.7	K^+	7.8
Br^-	0.04	Mg^{2+}	13.3
NO_3^-	7.1	Ca^{2+}	71.3
PO_4^{3-}	3.3	Mn	<0.04
SO_4^{2-}	99.4	Fe	<0.02

Table S7: Wastewater parameters recorded for a 24-hour mixed sample in the WWTP Lustnau on September 9, 2024. COD stands for "chemical oxygen demand".

**Parameter**	**Value**
COD	195 mg/L
P total	2.27 mg/L
Electr. conductivity	691 µS/cm
Temperature	18.8 °C
